# The in vivo study on the radiobiologic effect of prolonged delivery time to tumor control in C57BL mice implanted with Lewis lung cancer

**DOI:** 10.1186/1748-717X-6-4

**Published:** 2011-01-12

**Authors:** Xin Wang, Xiao-Peng Xiong, Jiade Lu, Guo-Pei Zhu, Shao-Qin He, Chao-Su Hu, Hong-Mei Ying

**Affiliations:** 1 Department of Radiation and Oncology, Cancer Center and Department of Oncology, Shanghai Medical College, Fudan University, Shanghai, PR China; 2 Department of Radiation and Oncology, Huashan Hospital, Fudan University, Shanghai, PR China; 3 Department of Nuclear Medicine, Renji Hospital, Shanghai Jiaotong University School of Medicine,Shanghai, PR China; 4 Department of Radiation Oncology, National University Hospital, Singapore, Singapore

## Abstract

**Background:**

High-precision radiation therapy techniques such as IMRT or sterotactic radiosurgery, delivers more complex treatment fields than conventional techniques. The increased complexity causes longer dose delivery times for each fraction. The purpose of this work is to explore the radiobiologic effect of prolonged fraction delivery time on tumor response and survival in vivo.

**Methods:**

1-cm-diameter Lewis lung cancer tumors growing in the legs of C57BL mice were used. To evaluate effect of dose delivery prolongation, 18 Gy was divided into different subfractions. 48 mice were randomized into 6 groups: the normal control group, the single fraction with 18 Gy group, the two subfractions with 30 min interval group, the seven subfractions with 5 min interval group, the two subfractions with 60 min interval group and the seven subfractions with 10 min interval group. The tumor growth tendency, the tumor growth delay and the mice survival time were analyzed.

**Results:**

The tumor growth delay of groups with prolonged delivery time was shorter than the group with single fraction of 18 Gy (P < 0.05). The tumor grow delay of groups with prolonged delivery time 30 min was longer than that of groups with prolonged delivery time 60 min P < 0.05). There was no significant difference between groups with same delivery time (P > 0.05). Compared to the group with single fraction of 18 Gy, the groups with prolonged delivery time shorten the mice survival time while there was no significant difference between the groups with prolonged delivery time 30 min and the groups with prolonged delivery time 60 min.

**Conclusions:**

The prolonged delivery time with same radiation dose shorten the tumor growth delay and survival time in the mice implanted with Lewis lung cancer. The anti-tumor effect decreased with elongation of the total interfractional time.

## Introduction

New radiation therapy techniques such as sterotactic radiosurgery and IMRT are featured with improving target dose conformity while minimizing radiation exposure to surrounding normal tissues [1-5]. However, these technologies require complex planning and delivery procedure thus a substantially prolongerd delivery time for each fraction.

According to radiobiological theory, the sublethal damage repair (SLDR) takes place not only between the frations but also during the irradiation. Cell killing tends to decrease with fraction delivery time increasing because of ongoing sublethal damage repair processes during dose delivery [6,7]. Therefore, it is reasonable to question whether the radiobiological effectiveness of intermittently delivered radiation over a prlonged time has the same biological effectiveness as those delivered continuously through conventional external beam radiation therapy (EBRT).

A number of studies have been published to investigate the impact of prolonged delivery time (such as used in IMRT) on biological effects at the cellular level and demonstrated that the total time to deliver a single fraction may have a significant impact on treatment outcome [8-15]. However, in-vivo study about the effects of treatment time and fractionation on tumor response and growth is lmited. To our knowledge, there were only two studies to evaluate the effect of proloned delivery time in SCCVII tumors using an in vivo-in vitro assay and they found that cell survival from clamped tumors tended to increase with elongation of the intervals, but not significantly [16,17]. They contributed the confiliction of the results in vitro and in vivo to reoxygenation. In this study, we attempt to evaluate the impact of prolonged fraction delivery time on tumor control and survival in C57BL mice implanted with Lewis lung cancer using growth delay assay and survival analysis.

## Methods

### Cell line and mice

The Lewis lung carcinoma (LLC) cell line purchased from the Division of Animals of FUDAN University was grown in RPMI 1640 (Gibco BRL, USA) supplemented with 10% fetal bovine serum, 100 μg/ml streptomycin and 100 U/ml penicillin. The cell line was incubated at 37°C in 5% CO^2 ^until near-confluency, harvested, washed, and counted using trypan blue exclusion. Female C57BL/6 mice weighing 16-18 g were used. For in vivo implantation, LLC cells were washed in Hanks' balanced salt solution (HBSS) and injected subcutaneously at 1 × 10^6 ^cells in 0.1 ml HBSS in the right hind limb of C57BL/6 mice. At 10 days after the injection, 48 mice with tumor diameter reached 0.8~1.0 cm were retained and randomly divided into 6 groups and radiated according to the predetermined schedule as described below. These animals were fed sterilized chow and tap water in accordance with Fudan University of Animal Resource Department protocols in a laminar flow room.

### Irradiation

Unanesthetized tumor-bearing mice were immobilized in a jig with customed modules with their legs fixed using adhesive tapes to receive a focal irradiation. Irradiation was delivered using ^60^Co therapy unit at a dose rate of 157.1cGy/min at room temperature. The dose was calibrated using a RAMTEC 1000 dosimeter. All mice were shielded with a specially designed lead apparatus to allow irradiation to the right hind limb. Mice were kept under these conditions until all irradiation finished.

### Radiation Schedule

To learn the radiobiological characteristics, 48 mice were randomly divided into 6 groups with 8 mice per group. The total dose was 18 Gy for all irradiated subjects except for the control. In addition to the control, 5 radiation schedules were used: (1) 1 fraction of 18 Gy without interruption, (2) 2 fractions of 9 Gy with interfraction intervals of 60 min, (3) 7 fractions of 2.57 Gy with interfraction intervals of 10 min, (4) 2 fractions of 9 Gy with interfraction intervals of 30 min, (5) 7 fractions of 2.57 Gy with interfraction intervals of 5 min.

### Assay

Tumor dimensions were measured with Vernier calipers once every two days. Tumor volumes were calculated as follows: Volume (mm^3^) = (H*W * L)/2 (H = height, W = width, and L = length of the tumor).

The tumor growth time (TGT) was defined as the time required for the initial tumor size to quadruple after the first day of treatment. The tumor growth delay time (TGDT) was defined as the TGT in each treated animal minus the mean TGT in the control group. The curves of tumor growth and calculated tumor growth delayed time among the 6 groups were compared.

The survival time of mice was also documented.

### Statistical analysis

Statistical differences among groups were determined using one-way analysis of variance (ANOVA). Kaplan-Meier survival curves were analyzed. The logrank test was used to assess if there were differences among the six groups in overall survival. Results are expressed as means ± SEM. The level of significance used for all comparisons was *P *< 0.05.

## Results

### The effect of the total interfraction interval time on the growth of tumor tissues

**Figure 1:**
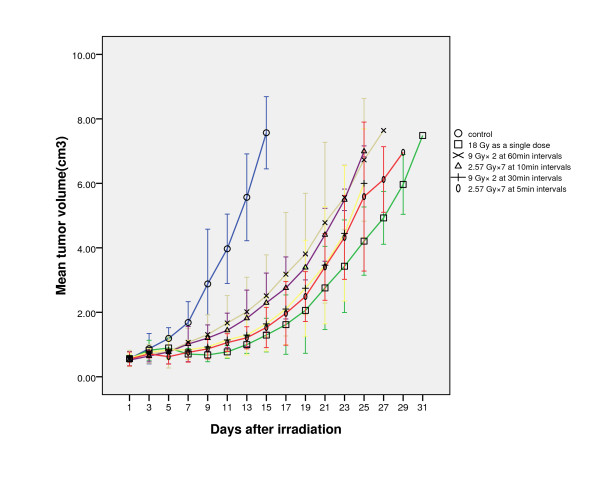
**The tumor growth curves of all groups treated with various radiation schedule and the control**. Tumor growth delay was significantly prolonged with the elongation of the total interfraction interval time.

Figure 1 shows the tumor growth curves of all groups treated with various radiation schedules and the control. Tumors in the control group demonstrated a rapid exponential growth. Tumors irradiated with a single fraction of 18 Gy produced the most significant growth delay. The other groups irradiated to 18 Gy with various fractions and intermittent time also demonstrated delay in tumor growth, which was significantly associated with interfractional intermittent time. The anti-tumor effect decreased with elongation of the total interfractional time. However, the fractionation dose was not associated with the tumor growth rate. (Table 1)

**Table 1:** Tumor growth time* compared to 18 Gy in a sigle fraction

Radiation schedule	TGT (days) ± SE	p value
Control	8.1 ± 0.6	<0.001
18 Gy as a single dose	19.9 ± 2.3	
9 Gy× 2 fractions at 60 min intervals	14.0 ± 1.8	<0.001
2.57 Gy×7 at 10 min intervals	14.3 ± 1.8	<0.001
9 Gy× 2 fractions at 30 min intervals	17.7 ± 2.5	0.034
2.57 Gy×7 at 5 min intervals	17.7 ± 2.6	0.037

* The tumor growth time (TGT) was defined as the time required for the initial tumor size to quadruple after treatment. Each date represents mean ± SE.

**Figure 2:**
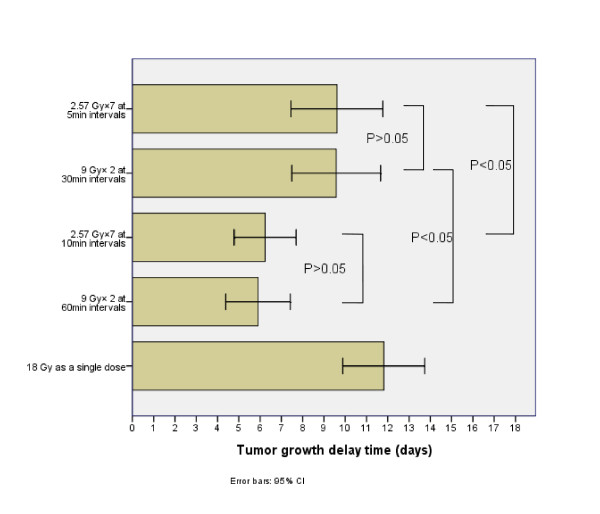
**The effects of different interfraction interval time on the TGDT**. The mean ± SE of tumor growth time in the group of the control is 8.09 ± 0.61 days. P < 0.05 as compared with the group irradiated with 18 Gy single fraction.

Figure 2 shows the tumor growth delay time (TGDT) of each group. The tumor growth delay of the groups with total interfraction interval time 30 min was higher than that of the groups with total interfraction interval time 60 min (P < 0.01). When the tumor growth delay of groups with the same total interfraction interval times was compared, there is no statistical significance (P > 0.05).

### The effect of the total interfraction interval time on survival time of the mice

As shown in table 2, the survival time of every irradiated group was longer than the control group; though the survival time of the four prolonged delivery time group was similar, the survival time of 18 Gy single fractions was much longer than the other four irradiated group.

**Table 2:** The survival time of each group (days)

Radiation schedule	survival time (days) ± SE	p value
Control	13.8 ± 2.4	<0.001
18 Gy as a single dose	28.8 ± 2.3	
9 Gy× 2 fractions at 60 min intervals	23.5 ± 3.7	0.011
2.57 Gy×7 at 10 min intervals	23.5 ± 3.7	0.004
9 Gy× 2 fractions at 30 min intervals	25.0 ± 2.9	0.026
2.57 Gy×7 at 5 min intervals	24.8 ± 2.8	0.027

The survival time of each group was compared with the group irradiated with 18 Gy single fraction. Each date represents mean ± SE. We also compared the last four groups by chi-square test, the F value is 0.428 and p value is 0.735. (data not shown in this table)

## Discussion

Our current knowledge of the effect of radiation on tumor growth were largely based on linear-quadratic (LQ) model, which was initially derived to fit experimental observations of the effects of dose and fractionation on cell survival, chromosomal damage and acute radiation effects. However, it was derived largely from in vitro rather than in vivo observations, thus does not consider that tumor response in vivo are affected by other effects such as the impact of ionizing radiation on the supporting tissues and the impact of the subpopulation of radioresistant clonogens. Therefore, our understanding of tumor response to different radiation fractionation or treatment time may be questionable for in vivo irradiation.

In the current study, the potential impact of prolonged fraction delivery time for a fixed total dose on the control of Lewis lung cancer implanted in C57BL mice was studied in order to investigate the effect of intermittent radiation exposure compared with that of continuous radiation exposure in vivo. The results of this in vivo study confirmed a rapid growth of tumor after prolonged intermittent time between fractions thus the total treatment time. However, the fraction size of radiation may not be a significant factor for tumor control.

The results of our in vivo study consisted with those previously reported studies which had focused to in vitro cell survival rates. Both in vitro radiobiological experiments and calculations based on the linear-quadratic model have shown greater cell survival rates for long 15-60 min compared to short 2-5 min fractional delivery times. Benedict et al irradiated several human GBM cell lines by the 6 MV γ rays of linear accelerator simulating intensity-modulated stereotactic radiosurgery. They divided the total doses into several fractions and the intervals ranged form 16 min to 3 hours. The results showed that the prolonged interval time will increase the survival fraction of the cells. A 40% increase in malignant glioma cell survival when the dose delivery schedule for a singlefraction 12 Gy irradiation was altered from 5 min of continuous irradiation to 60 min of intermittent irradiation were observed. Survival rates increased three-fold when the intermittent irradiation was stretched over 110 min. [8] Morgan and his colleague irradiated the tumor cancer cells simulating the IMRT plans. They delivered a total dose of 2 Gy to the cell lines over 2 min, 6 min and 20 min, and found that compared with the 2 min and 6 min group, the survival fraction of 20 min group increased significantly[9]. Wang et al reported total time to deliver a single fraction may have a significant impact on IMRT treatment outcome for tumors. They irradiated the human prostate cancer cells (the repair half-time is 16 min andα/β = 3.1 Gy) with different fraction delivery times in the range of 15-45 min. This study showed that for a prescription dose of 81 Gy in 1.8 Gy fractions, the EUD for prostate cancer decreased from 78 Gy for a conventional EBRT to 69 Gy for an IMRT with a fraction delivery time of 30 min; the TCP decreased almost 30% as well[10].

All the above-mentioned studies based on cell lines and were conducted under simplified in vitro conditions. The influence of other factors, such as proliferation, oxygen, and nutritional states in vitro is smaller than in tumors in vivo and repopulation, reoxygenation and bystander effects are obviously not considered.

In order to explore the biological effect of prolonged delivery time in vivo, Sugie et al conducted a study. In this study they used EMT6 and SCCVII tumors approximately 1 cm in diameter growing in the hind legs of syngeneic mice. Mice received whole body irradiation without anesthesia or physical restraint. Tumors were excised twenty hours after radiation and cell survival was determined by an in vivo-in vitro assay. They reported that no statistically significant decreases were observed by posing intervals between fractions in vivo. It was suggested that SLDR in vivo might be counterbalanced by other phenomena such as reoxygenation that sensitizes tumor cells to subsequent irradiation [16]. To explain the discrepancy between the in vitro and in vivo results, Tomita N et al conducted another in vivo study to evaluate the effect of intermittent radiation by using local irradiation to tumor-bearing legs and a tumor growth delay assay. They found that the fractionated groups had faster tumor regrowth than the continuously-irradiated control group, and the effect of radiation tended to decrease with elongation of interfraction intervals. In the present study, we studied the influence of the different fraction intervals to the mouse lewis cancer model. Our results are in consistent with those reported by Tomita although different experimental methods and anaimal models were used.

The discrepancy between the results in Sugie's study and ours may contribute to the technical problems associated with leg clamping and the magnitude and velocity of reoxygenation in tumors [17]. In Sugie's study, Mice received whole body irradiation without anesthesia or physical restraint. However, in Tomita's and our studies, unanesthetized tumor-bearing mice were immobilized in a jig with customed modules with their legs fixed using adhesive tapes to receive a focal irradiation. According to the study conducted by Shibamoto et al, when tumor-bearing mice were irradiated without anesthesia or physical restraint, the tumor had a hypoxic fraction of 5.4% [18]. Both anesthesia and immobilization of the tumor-bearing leg with adhesive tape produced significant increases in the hypoxic fraction (23 and 28%, respectively). Tomita's study showed that reoxygenation occurring within 5-15 min appeared to compensate for SLDR in SCCVII tumors. When tumor-bearing mice were immobilized, reoxygenation was limited and the magnitude of reoxygenation of hypoxic tumor cells might not be great enough to counterbalance SLDR, then the decrease of radiation effect occurred due to SLDR.

Although this study evaluated the radiation treatment time on the response and growth rate of tumor in vivo using tumor growth delay and survival analysis, a number of issues remain to be discussed. First, the heterogeneity of different tumor tissues have different capabilities of recovery from radiation [19], therefore the influence due to the prolonged delivery time may be different according to different tumor type. It is well known that the radiation sensitivity to low LET radiatoin is largely determined by sublethal damage repair, and dose-fractionation is an important factor for tumor killing and control [20], so the results obtained from the current series may not be applicable to all tumors. Second, the underlying mechanism of the differences in tumor response and delay of tumor growth due to treatment break time remains unknown. Third, our data provide a simplified estimate on the significance of prolonged delivery as a result of IMRT or radiosurgery. However, in reality the situation including the effects of instantaneous dose rate, beam-on time, and number, size and distribution of segments may be more complex. Moreover, the present study focused on tumor response only, and response and recovery of various normal tissues or organs from fractionated radiation over various irradiation time is complex and not addressed.

In general, our study demonstrated that prolonged delivery time significantly reduce the biological effect of radiation therapy in Lewis lung tumor. Treatment time may impact clinical outcome and should be recorded along with other established dosimetric parameters. These effects need to be confirmed in clinical trials and considered in treatment planning. Biologically, more reliabe experimental investigations using animal models based on human tumors are desirable. In addition, the underlying mechanism of tumor response and sublethal damage repair after radiation therapy should be investigated by examine multiple endpoints including cellular motility, metabolic activity and invasive capacities. Further studies are needed to establish more reliable radiobiological models to evaluate the relationship between interfaction intervals and the biologic effect of radiation.

## Competing interests

All authors declare there were no actual or potential conflicts of interest in this study.

## Authors' contributions

XW and XPX carried out the murine study, wrote the final version of the manuscript and contributed equally on this manuscript. CSH and SQH participated in the design of the study. HMY conceived of the study, and participated in its design and coordination. GPZ and JL provided some intellectual recommendation and reviewed the manuscript. All authors read and approved the final manuscript.
